# Editorial: Adipokines, batokines & cardiokines: crosstalk with metabolic organs

**DOI:** 10.3389/fendo.2024.1481180

**Published:** 2024-09-13

**Authors:** Noelia Martinez-Sanchez, Monica Imbernon

**Affiliations:** ^1^ Oxford Centre for Diabetes, Endocrinology and Metabolism, Radcliffe Department of Medicine, Medical Science Division, University of Oxford, Oxford, United Kingdom; ^2^ Blizard Institute, Barts School of Medicine and Dentistry, Queen Mary University of London, London, United Kingdom

**Keywords:** metabolic crosstalk, metabolic disease, adipokine, batokine, cardiokine, metabolic organs

Extensive findings have provided the importance of the crosstalk between metabolic tissues to modulate energy homeostasis and metabolism. Key metabolic organs, such as adipose tissues or heart, secrete different factors including peptides, metabolites, lipids or microRNAs that play an autocrine, paracrine and endocrine role, establishing communications with other cell types (immune cells) and key metabolic organs.

This Research Topic aimed to provide new insights into underlying mechanisms involved in the crosstalk between the heart, white and brown adipose tissues with the liver, muscle and central/peripheral nervous systems. The goal was to investigate whether this communication may impact the onset and progression of metabolic syndromes such as obesity, diabetes, metabolic dysfunction-associated steatotic liver disease (MASLD) and cardiovascular diseases.

This Research Topic contains four original articles, three reviews and one study protocol.

In the first original article, Pagnotta et al., investigate the paracrine actions of breast´s peritumoral adipocytes. They exposed white adipocytes´ cell line 3T3-L1 to conditioned media derived from human breast adipose tissue explants adjacent to tumours. The conditioned media promoted 3T3-L1 browning by reducing cell size, increased the number of small lipid droplets, reduced triglyceride content and upregulated thermogenic markers such as UCP1 or PGC1alpha were. The authors concluded that breast tumour environment, induces an autocrine and paracrine action of surrounding tumours´ adipocytes, promoting lipolysis and adipocyte browning through the secretion of different factors, including the action of other soluble factors secreted by the tumour cells.

The second original paper by Song et al., explored the adipokines derived from human subcutaneous adipose tissue and its clinical implication. The authors performed RNA sequence in subcutaneous and visceral adipose tissue samples from adult males together with analysis of selected serum proteins. They observed that GDF10 expression was higher in subcutaneous than in visceral adipose tissue and GDF10 serum levels were also increased in patients with obesity. GDF10 is a TGF-β family member expressed in bone but also in adipose tissue and it has been related to obesity in mice (1, 2). Therefore, they suggest GDF10 as a subcutaneous tissue´s adipokine related to the pathophysiology of obesity.

Continuing with the differences based on adipose tissue locations, Lempesis et al. explore the inflammatory signatures of abdominal (upper) and femoral (lower) subcutaneous adipose tissue in postmenopausal women with either normal weight or obesity. They analysed *in vivo* adipokines secretion using the arterio-venous balance technique, observing that upper and lower subcutaneous adipose tissue possesses distinct inflammatory signatures, which seem independent of the adipocyte size. Specifically, they detect that abdominal depots have a higher release of monocyte chemoattractant protein (MCP)-1 and lower gene expression of leptin, PAI-1 and tumour necrosis factor α compared with the femoral ones. In addition, in the upper depots, interleukin-6, PAI-1, and leptin gene expression were higher, while adiponectin and dipeptidyl-peptidase-4 gene expression were lower than in femoral adipocytes.

To demonstrate the importance of body crosstalk, Cisternas et al. explored the link between adipokines and cognitive function in ageing brain diseases, such as Alzheimer’s disease (AD). In this study, the authors studied the effect of adiponectin and resistin in control and AD mouse model (APP/PSN1), fed with a high fat-diet. They observed that in APP/PSN1, adiponectin administration induced a partial recovery in cognitive functions by 50% together with a reduction in aggregates of some pathogenic Amyloid β (Aβ) species. In contrast, resistin impaired Aβ pathology and increased oxidative stress. Altogether, the authors concluded that adiponectin and resistin contribute to AD pathology and their pharmacological modulation could be considered as a future treatment.

The three reviews included in this Research Topic explore different areas. First, Yang et al. focus on myostatin (MSTN), a hormone primarily produced by muscle cells, and its potential therapeutic role in metabolic syndrome including obesity, diabetes and hypertension. They discuss the role of MSTN in regulating cell metabolism, focusing on key organelles like mitochondria and essential processes such as autophagia. Furthermore, they explain MSTN’S involvement in mediating tissue crosstalk. Second, Perez-Arana et al., examine the novel roles of glucagon as insulinotropic in the pathophysiology of type 2 diabetes and the beneficial effects of glucagon after bariatric/metabolic surgery. Remarkably, the authors dedicate a section to discussing how different incretins influence and modulate glucagon secretion. Finally, in our latest review, Shi et al. highlight recent advancements in stem cell-based culture technology for studying treatments against diabetes and its complications. It delves into the novel opportunities offered by three-dimensional (3D) stem cell cultures and how the methodology that can improve both the quality of the culture and enhance cellular properties. Notably, they emphasize the significance of the paracrine activity of 3D-cultured adipose-derived mesenchymal stem cells (ASCs). The application of specific culture media together with various techniques, can enhance this paracrine potential, significantly impacting the cells’ characteristics.

Lastly, Cetin et al. presented a protocol for a randomized placebo-controlled clinical that aims to address the unknown positive link between palmitoleic acid (POA) and insulin sensitivity. POA, secreted by adipose tissue, appears to have beneficial effects by improving lipid profile and insulin sensitivity in the liver and muscle. The authors propose a double-blind placebo-controlled clinical trial test using pure exogenous POA in overweight and obese adult subjects.

They hypothesised that the use of pure POA supplementation will increase insulin sensitivity and decrease hepatic lipogenesis, demonstrating significant beneficial effects that other studies may have missed due to the use of POA supplements containing significant amounts of palmitic acid, which could mask these effects. The authors postulate that this could be a beneficial therapeutic approach for diabetes and immunometabolic disorders.

In this Research Topic, we highlighted the importance of the autocrine paracrine and endocrine communication between organs in the progression of different pathologies such as breast cancer, metabolic syndrome and Alzheimer’s disease.

We emphasized that for complex diseases involving multiple organs, novel approaches, such as 3D-based stem cell cultures, should be considered. Importantly, it is of relevance to promote research that deciphers the distinctive properties that hormones and molecules may drive in different organs and pathologies.

Altogether, this Research Topic aimed to provide an outlook of the latest studies where communication among tissues and organs is crucial to the pathophysiology of metabolic diseases.
